# The Use of Transcription Terminators to Generate Transgenic Lines of Chinese Hamster Ovary Cells (CHO) with Stable and High Level of Reporter Gene Expression

**Published:** 2015

**Authors:** N. B. Gasanov, S. V. Toshchakov, P. G. Georgiev, O. G. Maksimenko

**Affiliations:** Institute of Gene Biology, Russian Academy of Sciences, Vavilova Str., 34/5, Moscow, 119334, Russia

**Keywords:** Recombinant proteins, production of proteins in cell lines, transcription termination, insulators, CHO

## Abstract

Mammalian cell lines are widely used to produce recombinant proteins. Stable transgenic cell lines usually contain many insertions of the expression vector in one genomic region. Transcription through transgene can be one of the reasons for target gene repression after prolonged cultivation of cell lines. In the present work, we used the known transcription terminators from the SV40 virus, as well as the human β- and γ-globin genes, to prevent transcription through transgene. The transcription terminators were shown to increase and stabilize the expression of the *EGFP *reporter gene in transgenic lines of Chinese hamster ovary (CHO) cells. Hence, transcription terminators can be used to create stable mammalian cells with a high and stable level of recombinant protein production.

## INTRODUCTION

An increasing number of drugs are currently produced in cell culture bioreactors (first and foremost, those based on Chinese hamster ovary (CHO) cells) [1, 2]. However, the extremely high cost of the product is the main problem in manufacturing recombinant proteins in cell cultures. One of the ways to optimize the manufacturing process is to improve vectors for transgene generation, which allows one to significantly reduce the cost of manufacturing and maintenance of effective producer cell lines.

Transfection of linearized plasmid DNA has become the most common method used in bioengineering to generate cell lines for producing target proteins [3, 4]. This method can be employed to generate cell lines containing multiple copies of the expression vector that are usually inserted into one or, less frequently, several genomic sites. The cytomegalovirus (CMV) promoter, the SV40 early promoter, and strong housekeeping gene promoters, are typically used for transgene expression [5].

DNA sequences (usually the AT-rich ones) have been widely used since the early 1990s to enhance transfection efficiency and the stability of transgene expression; *in vitro *experiments demonstrated that these sequences interact with the matrix attachment region (MAR) [6-8]. The existing model assumes that the MAR elements interact with nuclear matrix proteins, thus reducing the dependence of the expression level of MAR-flanked genes on the negative effect of a chromatin environment.

Furthermore, known insulators are widely used to protect transgene transcription against repression and the negative effect of the surrounding genome [9-11]. The HS4 insulator (1.2 kbp) found at the border of the chicken β-globin locus is most typically used in bioengineering. Two copies of the HS4 insulator are usually inserted in the construct immediately downstream of the target gene. In some cases, either combinations of the known MAR and HS4 insulator are used or the HS4 core (500 bp) is multimerized [9]. These constructs enhance both the efficiency of transgene generation and the expression level of the transgene. However, the HS4 insulator is not equally efficient in all cell cultures and organisms.

Extended DNA fragments including housekeeping gene promoters (UCOE) [12, 13] and regulatory elements capable of blocking heterochromatin propagation [12, 14] are also used in bioengineering.

In general, it is fair to say that no universal regulatory elements with a comprehensible mechanism of action that could be efficiently used across all types of vector constructs intended for generating high-yield cell lines producing various proteins have been found yet. Even the strongest promoters are obviously expected to have mechanisms for suppressing excessive transcription. RNA interference is one of these mechanisms of transcription suppression [15, 16]. The suppression effects during transgene insertion are often associated with transcription through the transgene (e.g., transcription through enhancers inactivates their activity [17]). Long noncoding RNAs can also recruit repressive complexes to regulatory elements [18]. Based on the facts indicating that transcription occurring through regulatory elements plays a negative role in transgene expression, one can expect that transcription termination at transgene borders has a positive effect on the stabilization of transgene expression. Meanwhile, transcription is efficiently terminated only by some of the insulators under study [19].

Sequences of the well-studied transcription terminators from β- (βt) and ^G^γ- (γt) globin genes were used in this work to test the role of transcription termination in the protection of transgene expression [20, 21]. Two copies of the best-characterized HS4 (2×Ins) insulator from chicken β-globin locus were used as controls [22]. The transcription terminators were shown to be able to significantly increase the stability of reporter gene expression in cellular pools. When generating isolated stable cell lines, the constructs containing terminator regions were characterized by a higher level of reporter gene translation product.

## EXPERIMENTAL

**Creation of constructs**

In order to create a series of constructs, different sequences were inserted into the pEGFPN1 vector at the PciI restriction site located downstream of *EGFP *and upstream of cytomegalovirus promoter 640 bp away from the transcriptional start point: two tandem copies of the HS4 core insulator sequence from chicken β-globin locus (2×Ins) (476 bp), SV40 transcription terminator (SV40pA) (868 bp), transcription terminator from the human β-globin locus (βt) (1130 bp), and a combined element consisting of both βt and 2×Ins. A 1336-bp-long terminator from the human ^G^γ-globin gene (γt) was inserted into the construct βt_EGFP at the restriction site AflII to obtain the construct βt_ EGFP_γt.

**Creation of transfected cell lines**

Reporter gene expression was analyzed using Chinese hamster ovary (CHO-K1) cells, the cell culture most widely used in bioengineering to produce target proteins.

CHO-K1 cells were cultured on DMEM medium containing 10% of inactivated fetal bovine serum, 2mM *L*glutamine, 35 mg/L *L*-proline, and commercially available antibiotic Mycokill-AB (PAA Laboratories) at a working concentration. The cells were re-inoculated every 5 days at a concentration of 10^5^ cells/cm^2^ and a 1:20 dilution ratio. The cells were cultured at 37°C in an atmosphere of 5% CO_2_ and high moisture content. The cell culture was transfected with recombinant plasmids. Plasmids were linearized with the ApaLI restriction enzyme to ensure more efficient integration of the transgenic construct. The transfection protocol was as follows: The cells that reached 70–80% of the monolayer (8 × 10^4^ cells/cm^2^) were washed with a serumfree cell culture medium. The transfection mixture was prepared: 3–4 μg of linearized plasmid DNA was mixed with 375 μL of the serum-free cell culture medium. The commercially available transfection reagent Lipofectamine 2000 (with its amount calculated based on a ratio 3 μL of the reagent per 1 μg of plasmid DNA) was mixed with the same amount of the serum-free cell culture medium in a separate test tube. The solutions were combined and incubated at room temperature for 30–40 min. The washed cells were coated with the transfection mixture. The transfection mixture was replaced with a serum-containing DMEM culture medium 4–6 h after the transfection had been initiated.

The level of reporter gene expression was assessed according to the fluorescence intensity on day 2 after transfection (36–48 h) by flow cytometry on a MACSQuant Analyzer VYB (MiltenyiBiotec). Nontransfected CHO-K1 cells were used as negative control.

**Cytofluorometric analysis**

Prior to the cytofluorometric analysis, the cells were washed with phosphate buffered saline (PBS), treated with trypsin, and removed from the Petri dishes. They were subsequently washed twice to remove trypsin and thoroughly re-suspended in phosphate buffered saline. The resulting suspension (106 cells per mL of PBS) was transferred into 5-mL round-bottom test tubes.

The voltage in the flow cytofluorometer channels was selected so as not to take into account the autofluorescence of nontransfected cells. After calibration, all the samples were measured at a constant voltage. Thus, if EGFP fluorescence was detected, we counted the cells that emerged in the range of values above 10 on the logarithmic scale for a proper channel and measured all the quantitative values of the gene expression level.

**Maintenance of the transfected cell pools**

After the transfection, cell pools were cultured according to the above protocol. Transgene-free cells were removed by selecting the transfected cell pools using the commercially available antibiotic Geneticin (Invitrogen) at a concentration of 800 μg/mL. Since suppression of the transcriptional activity of the transgene reduces production of the antibiotic-resistance gene with time, the antibiotic concentration needs to be gradually reduced to 200 μg/mL after the cells have been cultured for 77 days.

**Creation of individual clones**

Pools of cultured cells were removed from the plates and diluted in 10 mL of the culture medium. Cell concentration was then determined using a Scepter automated cell counter (Millipore); the cells were diluted so that 1 mL of the medium contained 2–3 cells. The resulting suspension was transferred into 24-well plates (1 mL per well).

After cultivation in a DMEM medium containing antibiotic Geneticin at a concentration of 800 μg/μL for two weeks, we performed a cytofluorometric analysis of the clones that survived. The resulting cell lines were further maintained, and the expression level of EGFP was determined according to the procedure for transfected cell pools. EGFP fluorescence intensity was measured every 15 days.

## RESULTS AND DISCUSSION

**Fig. 1:**
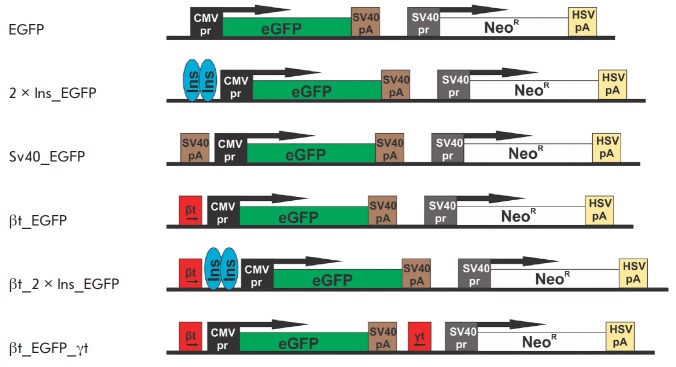
Schemes of the constructs used to test DNA elements in the CHO-K1 cell culture. EGFP is the control plasmid pEGFPN1 containing no DNA elements. 2×Ins_EGFP is the control construct with two copies of the insulator from chicken β-globin locus inserted upstream of the cytomegalovirus promoter. SV40_EGFP, βt_EGFP, and βt_2×Ins_EGFP are the constructs with transcription terminators inserted upstream of the cytomegalovirus promoter. βt_EGFP_γt is the construct with transcription terminators surrounding the *EGFP *reporter gene. Cytomegalovirus and virus SV40 promoters are shown as black and gray squares, respectively; transcription terminators are shown as brown (SV40), yellow (HVS thymidine kinases), and red (*β*- and*γ*-globin genes) squares. The *EGFP *and neomycin resistance genes are shown as green and white rectangles, respectively; arrows indicate transcription direction. Insulators are shown as light blue ovals

The enhanced green fluorescent protein (EGFP) gene under control of the CMV promoter was used as a reporter gene when studying the potential role of transcription terminators in stabilizing the transgene expression level in CHO cells. All the experimental constructs were compared to the control, the plasmid pEGFPN1 (Clontech) that contained the *EGFP *gene under the control of the CMV promoter (CMVpr) and SV40 transcriptional terminator (SV40pA) (EGFP construct in *Fig. 1*). In addition to the reporter gene, this plasmid contained the Neomycin resistance gene (NeoR) under the control of the SV40 promoter (SV40pr) and transcription terminator of the herpes simplex virus thymidine kinase gene (HSVpA). In this case, the Neomycin resistance gene is needed for selecting transfected cells.

The well-studied transcription terminators of the β-globin gene (βt) and SV40pA were used to assess the effect of termination of transcription initiated downstream of the inserted transgene on reporter gene expression. In the constructs (SV40_EGFP and βt_EGFP), these terminators were inserted in forward orientation immediately upstream of the CMV promoter (*Fig. 1*). In order to completely isolate the reporter gene from transcription initiated in the surrounding chromatin, we created a derivative construct βt_EGFP (*Fig. 1*), which contained, in addition to the β-globin terminator, the transcription terminator from the Gγ-globin gene (βt_EGFP_γt) inserted in reverse orientation at the 3’- end of the reporter gene. In this construct, the reporter gene is protected on both sides against transcription initiated from the surrounding chromatin.

In order to compare the efficiency of transcription terminators with the currently known regulatory elements stabilizing transgene expression in CHO cells, we used an element consisting of two copies of the HS4 core insulator sequence inserted immediately downstream of the CMV promoter (2×Ins_EGFP, *Fig. 1*). Finally, a βt_2×Ins_EGFP construct with the globin terminator inserted downstream of the two copies of the insulator was generated to study the cooperative effect of two regulatory elements with different functions: the insulator and transcription terminator (*Fig. 1*).

The levels of reporter gene expression during transfection of different construct variants were measured using a CHO-K1 cell culture, which is most widely used in bioengineering to produce target proteins. Transfection was performed using the conventional procedure employing liposomes.

**Fig. 2:**
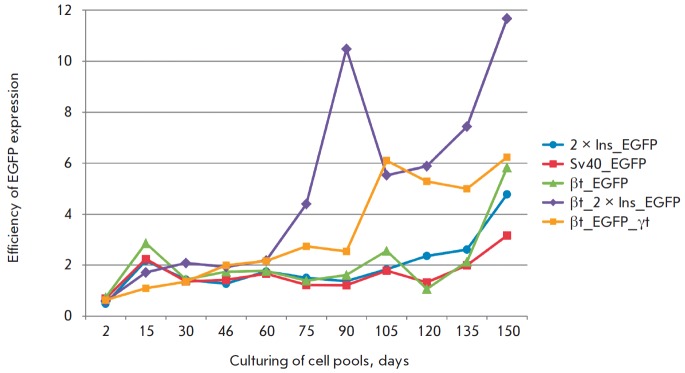
Analysis of the expression level of the *EGFP *reporter gene in cellular pools transfected with constructs. The histogram shows the results of measuring the fluorescence of stable cellular pools using flow cytofluorometry every 15 days. The Y axis shows the efficiency of *EGFP *expression determined as the ratio between the percentage of *EGFP*expressing cells in the pool of cells carrying the construct with this element (2×Ins_EGFP, SV40_EGFP, βt_EGFP, βt_2×Ins_EGFP, βt_EGFP_γt) and the percentage of *EGFP*-expressing cells with the control plasmid (EGFP). The mean expression level of *EGFP *in the pool of cells at a certain moment was assessed using the percentage of cells with a fluorescence intensity above 10 on a logarithmic scale in the corresponding channel. Each curve in the histogram represents an individual construct

The levels of reporter gene expression were estimated every 15 days using flow cytofluorometry to determine the degree of transgene suppression in the cell pools transfected with the control (pEGFP_N1 and 2×Ins_EGFP) and experimental (SV40_EGFP, βt_ pEGFP, βt_2×Ins_EGFP, and βt_EGFP_γt) constructs (*Fig. 2*). The mean expression level of the *EGFP *gene in the cell pool at a certain moment was determined according to the percentage of cells with a fluorescence intensity higher than 10 on the logarithmic scale (i.e., cells generating a significant amount of this protein). The ratio between the percentage of *EGFP*-expressing cells in the pool of cells carrying the construct with this element and the percentage of *EGFP*-expressing cells with the control plasmid (EGFP) was an indicator of the efficiency of a particular regulatory element in achieving a high and stable level of protein production.

The results of determining the *EGFP *expression level during culturing of the total population of transfected cells for 150 days (which corresponds to ~ 30 passages) indicate that the use of a transcription terminator downstream of the target gene promoter significantly enhances the efficiency of protein production during prolonged cultivation: over threefold for terminator SV40 and fivefold for terminator βt by the end of the experiments (*Fig. 2*). The regulatory element consisting of two copies of the insulator also had a similar effect.

The increase in the level of EGFP expression was higher in the pool of cells transfected with βt_EGFP_γt plasmid, where the reporter gene is surrounded by transcription terminators (*Fig. 2*). Thus, protection of the reporter gene (on both sides) against transcription initiated outside the transgene enhances stability and expression efficiency. Finally, the best results (a 12-fold increase) were achieved when the transcription terminator and two copies of the insulator were combined in the plasmid βt_2×Ins_EGFP (*Fig. 2*). A conclusion can be drawn that two regulatory elements with different mechanisms of action exhibit an additive effect on the stability and efficiency of regulatory gene expression.

Recombinant proteins are produced in actual practice by selecting stable cell lines generated from a single transfected cell and, therefore, having a heterogeneous genotype, which allows one to eliminate the effect of various negative factors and isolate the most efficient clone with the optimal site of construct integration in the genome.

Individual cellular clones were obtained using the limiting dilution technique after culturing total cell populations for 30 days. The median cell distribution over the fluorescence intensity was the main qualitative indicator of expression activity of the reporter gene.

The following individual clones were initially obtained: 10 clones containing the EGFP construct, 17 clones containing the 2×Ins_EGFP construct, 10 clones containing the SV40_EGFP construct, and 10 clones containing the βt_EGFP construct. Among those, two clones with the EGFP construct, five clones with the 2×Ins_EGFP, five clones with the SV40_EGFP construct, and six clones containing the βt_EGFP construct were characterized by a sufficiently high level of *EGFP* expression.

**Table:** Results of analysis of the temporal expression profile of the EGFP gene in individual cell clones

Clone	Fluorescence intensity (the median distribution), 30 days	Fluorescence intensity (the median distribution), 90 days	Decrease in expression activity	Mean value
EGFP #1	23.71	2.35	0.1	0.1
EGFP #2	16.11	1.7	0.11	
2xIns_EGFP #1	69.78	9.73	0.14	0.13
2xIns_EGFP #2	116.52	3.59	0.03	
2xIns_EGFP #3	103.66	16.6	0.16	
2xIns_EGFP #4	33.08	5.99	0.18	
2xIns_EGFP #5	77.74	10.55	0.14	
SV40_EGFP #1	67.93	13.1	0.19	0.76
SV40_EGFP #2	339.82	82.79	0.24	
SV40_EGFP #3	50.25	134.45	2.68	
SV40_EGFP #4	7.04	2.37	0.34	
SV40_EGFP #5	3.31	1.24	0.37	
βt_EGFP #1	78.44	15.54	0.2	2.07
βt_EGFP #2	79.86	19.63	0.25	
βt_EGFP #3	76.35	14.46	0.19	
βt_EGFP #4	2.19	13.34	6.09	
βt_EGFP #5	15.75	56.74	3.6	

^1^Code of the amino acid sequence in the Swiss-Prot database of protein structures (www.uniprot.org).

^2^Classification into CT Group I and II is based on the presence of either two Pro (Group I) or a single Pro (group II) residues in the loop I sequence.

It should be mentioned that clones containing the control construct initially showed a much lower level of *EGFP *expression compared to those containing the 2×Ins_EGFP, SV40_EGFP, and βt_EGFP constructs (*Table 1*).

The level of *EGFP *expression in stable cell clones containing the EGFP and 2×Ins_EGFP constructs decreased approximately tenfold after cultivation for 2.5 months. The average expression activity of the clones containing the SV40_pEGFP and βt_pEGFP constructs was ~ 75% of the initial value.

The results of measuring stable cell pools demonstrated that the most efficient cells characterized by a stable expression of the reporter gene were obtained after culturing for ~ 90 days. Hence, in order to study the clones generated from a stabilized cell population in more detail, we repeated the procedure of generating individual cell clones from total populations using the limiting dilution procedure after culturing stable pools for 90 days.

**Fig. 3:**
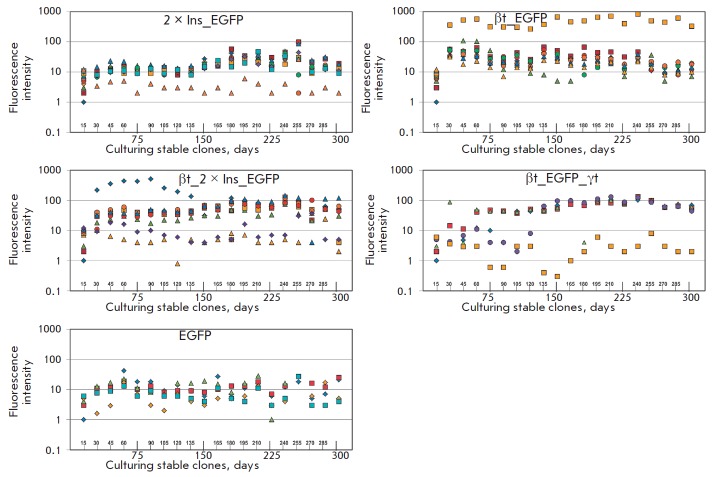
Analysis of *EGFP *reporter gene expression in stable cell clones generated using the transfected cell pools. The histograms show the changes in the fluorescence intensity of stable cell clones recorded every 15 min by flow cytofluorometry. The X axis shows the measurement intervals. The Y axis shows the logarithmic scale of EGFP fluorescence intensity, which was determined as the distribution median. The mean expression level of *EGFP *in the cellular pool at a certain moment was assessed as a value being directly proportional to EGFP fluorescence intensity. Each point in the histogram represents an individual cell clone

Identically to the previous experiment, the median cell distribution over the fluorescence intensity was the main quantitative indicator of the activity of reporter gene expression. Twelve individual clones with the EGFP, 2×Ins_EGFP, βt_EGFP, βt_2×Ins_EGFP, and βt_EGFP_γt constructs were initially obtained. Some clones died by the end of the experiment (culturing the cell clone for 300 days); hence, we report the data for five clones containing the EGFP construct, 12 clones containing the 2×Ins_EGFP construct, nine clones containing the βt_EGFP construct, nine clones containing the βt_2×Ins_EGFP construct, and five clones containing the βt_EGFP_γt construct (*Fig. 3*). The measurements demonstrate that the resulting EGFP clones are characterized by a stable, but extremely low, level of fluorescence (the average values fluctuate around 10). The levels of fluorescence are slightly higher for 2×Ins_EGFP clones: only one clone has low activity, while the activities of the remaining ones lie in a range between 10 and 100. βt_EGFP clones are characterized by even higher average fluorescence values compared to 2×Ins_EGFP clones. Furthermore, one of the βt_EGFP clones exhibited ultra-high levels of fluorescence (between 10 and 1000). The βt_2×Ins_EGFP clones unexpectedly divide into two groups: one group (two clones) was characterized by low levels of fluorescence comparable to those of the clones containing the EGFP construct, while the second group was characterized by much higher values (about 100), which were on average higher than those for the 2×Ins_EGFP and βt_EGFP clones. This discrepancy in results probably arises from the fact that we used a more complex combination of regulatory elements (instable in certain genomic regions). Similar activity was observed for βt_EGFP_γt clones. Among them, one clone was characterized by an extremely low level of fluorescence, while the other ones exhibited a high level of fluorescence (about 100). The results of this experimental series can be used to draw a conclusion that transcription terminators are very efficient in establishment and maintenance of a high level of target protein production. The combined element (terminator attached to the insulator) and the variant containing the reporter gene surrounded by terminators are characterized by a higher efficiency of target protein production. However, these constructs require a more careful selection of clones, since some clones turned out to be ineffective due to some unknown reasons.

## CONCLUSIONS

It has been demonstrated that transcription terminators, which can potentially isolate the transgene from transcriptional signals, are capable of maintaining the transgene transcription level stably high for an appreciably long period of time when culturing CHO-K1 cell lines. The terminator was found to maintain a stable level of transgene expression more effectively compared to the insulator. Furthermore, a combination of the transcription terminator and the insulator exhibits an additive effect, which enhances and stabilizes transgene expression.

## Abbreviations

RB: recombinant protein
CHO: Chinese hamster ovary cells
UTR: untranslated region of the gene
kbp: thousands of nucleotide base pairs
S/MAR: DNA regions associated with nuclear matrix proteins
insulators: regulatory elements that block the interaction between the enhancer and the promoter
UCOE: regulatory elements containing strong promoters of the housekeeping genes
STAR: regulatory elements protecting against HP1-dependent repression
EGFP: enhanced green fluorescent protein
CMV: cytomegalovirus
SV40: simian virus 40
HSV: herpes simplex virus

## References

[1] Kim J.Y., Kim Y.G., Lee G.M. (2012). Appl. Microbiol. Biotechnol..

[2] Hacker D.L., De Jesus M., Wurm F.M. (2009). Biotech. Advances..

[3] Khan K.H. (2013). Adv. Pharm. Bull..

[4] Browne S.M., Al-Rubeai M. (2007). Trends Biotech..

[5] Lai T., Yang Y., Ng S.K. (2013). Pharmaceuticals (Basel)..

[6] Kim J.M., Kim J.S., Park D.H., Kang H.S., Yoon J., Baek K., Yoon Y. (2004). J. Biotech..

[7] Girod P.A., Zahn-Zabal M., Mermod N. (2005). Biotech. Bioeng..

[8] Harraghy N., Gaussin A., Mermod N. (2008). Current Gene Therapy.

[9] Recillas-Targa F., Valadez-Graham V., Farrell C.M. (2004). BioEssays..

[10] Kwaks T.H., Otte A.P. (2006). Trends Biotechnol..

[11] Maksimenko O.G., Deykin A.V., Khodarovich Y.M., Georgiev P.G. (2013). Acta Naturae..

[12] Palazzoli F., Bire S., Bigot Y., Bonnin-Rouleux F. (2011). Nat. Biotechnol..

[13] Antoniou M., Harland L., Mustoe T., Williams S., Holdstock J., Yague E., Mulcahy T., Griffiths M., Edwards S., Ioannou P.A. (2003). Genomics..

[14] Kwaks T.H.J., Barnett P., Hemrika W., Siersma T., Sewalt R.G., Satijn D.P., Brons J.F., van Blokland R., Kwakman P., Kruckeberg A.L. (2003). Nat. Biotechnol..

[15] Saxena A., Carninci P. (2011). BioEssays..

[16] Martianov I., Ramadass A., Barros A.S., Chow N., Akoulitchev A. (2007). Nature.

[17] Erokhin M., Davydova A., Parshikov A., Studitsky V.M., Georgiev P., Chetverina D. (2013). Epigenetics Chromatin..

[18] Khalil A.M., Guttman M., Huarte M., Garber M., Raj A., Rivea M.D., Thomas K., Presser A., Bernstein B.E., van Oudenaarden A. (2009). Proc. Natl. Acad. Sci. USA..

[19] Silicheva M., Golovnin A., Pomerantseva E., Parshikov A., Georgiev P., Maksimenko O. (2010). Nucleic Acids Res..

[20] Nojima T., Dienstbier M., Murphy S., Proudfoot N.J., Dye M.J. (2013). Cell Rep..

[21] Plant K.E., Dye M.J., Lafaille C., Proudfoot N.J. (2005). Mol. Cell. Biol..

[22] Hanawa H., Yamamoto M., Zhao H., Shimada T., Persons D.A. (2009). Mol. Therapy..

